# Next-Generation Sequencing Analysis of the *Tineola bisselliella* Larval Gut Transcriptome Reveals Candidate Enzymes for Keratin Digestion

**DOI:** 10.3390/genes12081113

**Published:** 2021-07-22

**Authors:** Michael Schwabe, Sven Griep, Henrike Schmidtberg, Rudy Plarre, Alexander Goesmann, Andreas Vilcinskas, Heiko Vogel, Karina Brinkrolf

**Affiliations:** 1 Department of Bioresources, Fraunhofer Institute for Molecular Biology and Applied Ecology, Ohlebergsweg 12, 35392 Giessen, Germany; michael.schwabe@ime.fraunhofer.de (M.S.); andreas.vilcinskas@agrar.uni-giessen.de (A.V.); 2 Bioinformatics and Systems Biology, Justus Liebig University Giessen, Heinrich-Buff-Ring 58, 35392 Giessen, Germany; sven.griep@computational.bio.uni-giessen.de (S.G.); alexander.goesmann@computational.bio.uni-giessen.de (A.G.); 3 Institute for Insect Biotechnology, Justus Liebig University Giessen, Heinrich-Buff-Ring 26-32, 35392 Giessen, Germany; henrike.schmidtberg@agrar.uni-giessen.de; 4 Bundesanstalt für Materialforschung und -prüfung (BAM), Unter den Eichen 87, 12205 Berlin, Germany; ruediger.plarre@bam.de; 5 Entomology Department, Max-Planck Institute for Chemical Ecology, Hans-Knoell-Strasse 8, 07745 Jena, Germany; hvogel@ice.mpg.de

**Keywords:** gene expression, RNA-Sequencing, transcriptomics, *Tineola bisselliella*, keratin, insect biotechnology

## Abstract

The clothes moth *Tineola bisselliella* is one of a few insects that can digest keratin, leading to the destruction of clothing, textiles and artwork. The mechanism of keratin digestion is not yet fully understood, partly reflecting the lack of publicly available genomic and transcriptomic data. Here we present a high-quality gut transcriptome of *T. bisselliella* generated from larvae reared on keratin-rich and keratin-free diets. The overall transcriptome consists of 428,221 contigs that were functionally annotated and screened for candidate enzymes involved in keratin utilization. As a mechanism for keratin digestion, we identified cysteine synthases, cystathionine β-synthases and cystathionine γ-lyases. These enzymes release hydrogen sulfite, which may reduce the disulfide bonds in keratin. The dataset also included 27 differentially expressed contigs with trypsin domains, among which 20 were associated with keratin feeding. Finally, we identified seven collagenases that were upregulated on the keratin-rich diet. In addition to this enzymatic repertoire potentially involved in breaking down keratin, our analysis of poly(A)-enriched and poly(A)-depleted transcripts suggested that *T. bisselliella* larvae possess an unstable intestinal microbiome that may nevertheless contribute to keratin digestion.

## 1. Introduction

The common clothes moth (*Tineola bisselliella*) is one of only a few eukaryotic organisms that can digest keratinaceous materials such as wool and feathers. Among insects, this ability is limited to some bird lice and the larvae of certain carpet beetles (e.g., *Attagenus unicolor*, *Trox* sp.) and moths (e.g., *Tinea pellionella*, *Hofmannophila pseudospretella*) [1,2]. The clothes moth, a synanthropic species that lives mostly in urban areas, is the best-known example [3]. While adult clothes moths do not feed, their larvae are classed as industry and museum pests due to their voracious feeding behavior. Although few eukaryotic organisms can feed on keratin-rich materials, keratin is one of the most abundant structural proteins in nature. It is a waste product from poultry farms, slaughterhouses and the leather industry, and comes in many forms including feathers, wool, horn and hooves. Global feather waste ranges from 800,000 to 65,000,000 tons per year [4,5]. This waste is usually buried in landfills, burned for energy recovery or processed into compost, thus wasting valuable amino acids while contributing to environmental pollution [4,6,7]. Keratin is a fibrous protein that is highly crosslinked by intermolecular and intramolecular disulfide bonds, reflecting the 7–20% content of cysteine residues in the keratin polypeptide [8,9]. Keratin degradation therefore requires initial sulfitolysis to break the disulfide bonds and generate a linear polypeptide, which is then accessible for proteolysis by peptidases.

The larvae of *T. bisselliella* have been investigated to understand the mechanism of keratin degradation. Growth on keratin-rich diets depends on B vitamins, other vitamins and sterols [10]. For this reason, *Tineola* larvae usually feed on materials soiled with dirt and sweat, and yeast is added to the diet under standard laboratory rearing conditions [10]. The larval gut has a high pH (up to 10) and a very low redox potential (down to −300 mV). These conditions appear to be diet-independent and are not uncommon within the order Lepidoptera, but are thought to be required for keratin degradation [11,12]. In addition, free hydrogen sulfide is present in the gut when larvae consume keratin-rich food [13,14,15]. Accordingly, the enzyme cocktail in the *Tineola* larval gut includes a highly active peptidase that is largely insensitive to thiol compounds [11] as well as other enzymes that release hydrogen sulfide, such as cysteine desulfhydrase [16], cystine reductase, and glutathione reductase [17]. Other studies have identified cysteine lyase and cysteine desulfhydrase but found no evidence of cystine reductase activity [13,18]. A transcriptome-level study quantifying expressed sequence tags (ESTs) in the *Tineola* larval gut, reported the prevalence of ESTs with homology to trypsin-like enzymes and serine-type endopeptidases [2]. The authors did not detect subtilisin-type proteases as found in most keratinolytic prokaryotes. Some studies have shown that keratin degradation in *T. bisselliella* differs from the mechanism in *Trox* beetles (*Trogidae*) [2] and dermestid beetles [19], which also feed on keratin diets.

Most keratinases described thus far originate from bacteria, but the *T. bisselliella* larval gut is not inhabited by a persistent microbial community even though some recently identified species of *Bacillus* may contribute to keratin degradation [20,21,22]. Keratin degradation mechanisms have been reported for bacteria and fungi [23]. The term ‘keratinase’ is used for single enzymes as well as enzyme complexes. Most bacterial keratinases are subtilisin-type (serine-type) proteases, but some are metallo-type or serine-metallo-type enzymes [23,24]. Keratinolytic fungi reduce keratin using a complex mixture of enzymes including a disulfide reductase and free sulfide transporters [23].

The mechanism of keratin digestion in *T. bisselliella* larvae is not fully understood. Their intestinal transcriptome/proteome is not completely described and a genome is not yet available. Despite the identification of keratinolytic bacteria in the clothes moth, microbial abundance levels in larval guts are either low or their distribution among tested samples is patchy. We therefore cannot rule out the possibility that *T. bisselliella* larvae produce their own keratinolytic enzymes. Accordingly, we prepared a de novo assembly of the *T. bisselliella* larval gut transcriptome on different diets and identified the differentially expressed genes. One of the diets was based on feathers and rich in keratin, while the other diet was keratin-free, consisting of insect carcasses. These feeding conditions were used to analyze diet-specific enzymes that are involved in the digestion of keratin, provided that the regulation of digestive enzymes is food-dependent. We confirmed that known keratinolytic enzymes were represented in the transcriptome dataset, but identified some new candidates with and without diet dependent regulation that contribute to a more detailed understanding of keratin utilization in *T. bisselliella* larvae.

## 2. Materials and Methods

### 2.1. Animal Rearing and Sample Preparation

Specimens of *T. bisselliella* were obtained from the Bundesanstalt für Materialforschung und -prüfung (BAM, Berlin, Germany), where they were reared on keratin-rich and keratin-free diets at 27 ± 2 °C and 70 ± 5% relative humidity. The keratin-rich diet consisted of goose feathers. Feathers were stored at −18 °C for at least 3 days before washing with a yeast suspension (5% baker’s yeast in water) and drying at 65 °C for at least 72 h. The feather diet was stored at −18 °C until use. The keratin-free diet consisted of insect carcasses. *T. bisselliella* was grown for several generations on both diets prior to sample generation.

**Figure 1:**
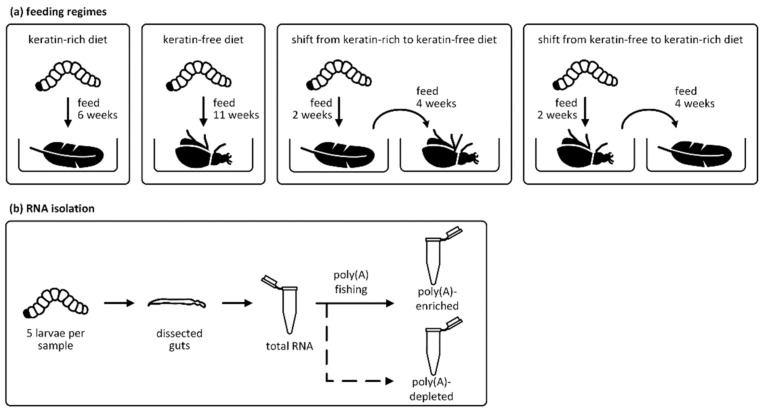
(**a**) Animal rearing and sample preparation. *Tineola bisselliella* larvae were grown on a keratin-rich diet based on goose feathers or a keratin-free diet composed of insect carcasses. Four different sample types were generated: (1) larvae grown exclusively on the keratin-rich diet for 6 weeks; (2) larvae grown exclusively on the keratin-free diet for 11 weeks; (3) larvae grown on the keratin-rich diet for 2 weeks and transferred to the keratin-free diet for another 4 weeks; and (4) larvae grown on the keratin-free diet for 2 weeks and transferred to the keratin-rich diet for another 4 weeks. (**b**) RNA isolation and fractionation. Guts were dissected from five larvae for each replicate. Total RNA was processed by poly(A) fishing to produce poly(A)-enriched and poly(A)-depleted fractions.

We sampled larvae from four different diet groups: (1) larvae grown on a feather diet until the final growth stage (45 days); (2) larvae grown on insect carcasses until the final growth stage (~75 days); (3) larvae grown on the feather diet for 14 days and then transferred to the insect carcass diet until the final growth stage (another 28 days); and (4) larvae grown on the insect carcass diet for 16 days and then transferred to the feather diet until the final growth stage (28 days) (Figure 1a). Larvae from the final growth stage in each group were extracted from their cocoons, washed under tap water three times and transferred to PBS (4°C) for gut dissection. Undamaged guts were separated from the fat body tissue and trachea, and were washed three times with fresh PBS (4°C). For each sample/replicate, five guts were pooled, directly transferred to tissue lysis buffer from the InnuPREP RNA Mini Kit (Analytik Jena, Jena, Germany) and shock frozen at −80 °C before RNA extraction.

### 2.2. RNA Isolation and Sequencing

RNA was extracted using the InnuPREP RNA Mini Kit following the manufacturer’s protocol. Samples were homogenized in a TissueLyzer (Qiagen, Hilden, Germany) using ceramic beads. DNA was digested with TurboDNase (Thermo Fisher Scientific, Langenselbold, Germany) and total RNA was purified using RNA Clean and Concentrator 5 (Zymo Research, Irvine, CA, USA). The integrity of all RNA samples was verified using an Agilent 2100 Bioanalyzer and an RNA 6000 Nano Kit (Agilent Technologies, Santa Clara, CA, USA). RNA quantity was determined using a Nanodrop ND-1000 UV/Vis spectrophotometer (Thermo Fisher Scientific).

Total RNA was separated into poly(A)-enriched and poly(A)-depleted mRNA fractions (Figure 1b). The rRNA fraction was depleted using the Ribo-Zero rRNA Removal Kit (Illumina, Berlin, Germany) and poly(A)-containing mRNAs were isolated using the TruSeq RNA Library Prep Kit (Illumina, Berlin, Germany) leaving the remaining poly(A)-depleted fractions. The 18 matching poly(A)-enriched and poly(A)-depleted samples were sequenced by the Max Planck Genome Center Cologne (MPGCC) on an Illumina HiSeq3000 Genome Analyzer platform, generating paired-end (2 × 150 bases) reads.

### 2.3. Sequence Data Quality Control and Transcriptome Assembly

Read quality was assessed with FastQC v0.11.5 [25]. Low-quality regions and adapters were trimmed with Trimmomatic v0.36 [26] using the following parameters: ILLUMINACLIP:illumina_adapters.fa:2:30:1, SLIDINGWINDOW:4:15, MINLEN:50. Quality-filtered reads were assembled de novo with Trinity v2.4.0 [27] using the default *k*-mer size of 25, and assembly statistics were calculated using TrinityStats.pl in the Trinity package. All quality-filtered reads were mapped using Bowtie2 v2.3.4.3 in end-to-end mode (--end-to-end) against the transcriptome assembly [28]. Contigs were classified as those expressed in (i) poly(A)-enriched fractions, (ii) poly(A)-depleted fractions or (iii) not expressed. We used the Benchmarking Universal Single-Copy Orthologs tool from BUSCO v 3.0.2 [29,30] to evaluate the completeness of the de novo transcriptome using the classified contigs as input. All BUSCO databases included in the analysis (e.g., Insecta_odb9; see Supplementary Table S1) were downloaded from the BUSCO homepage.

### 2.4. Gene Finding and Functional Annotation

Open reading frames (ORFs) were predicted using TransDecoder v5.0.2 [31]. DIAMOND v0.9.17 [32] and the BLAST suite (BLAST v2.6.0+) [33,34] were then used to assign gene functions to contigs with ORFs using the TransDecoder results as input sequences and the MEROPS [35], UniProt [36] and NCBI nr [37] databases. To assign functions to contigs without predicted ORFs, contigs were aligned to the NCBI nt database [37]. Hits were retained where the subject or query coverage was ≥70% and the identity was ≥40% [38]. The query and subject coverage requirement should ensure that nearly all functionally relevant domains are present on the contig. Pfam domains were analyzed using hmmscan (HMMER suite v3.1b2) [39] with the Pfam-A database v31.0 [40] as a reference. Each hit was exclusively classified into one of three categories: (i) complete (calculated domain coverage ≥90%), (ii) partial (calculated contig coverage ≥90%) and (iii) fragmented (all others). For all categories the e-value cutoff was ≤0.0001.

### 2.5. Identification of Proteins with Putative Keratinolytic Activity

To identify contigs with similarities to sequences with known keratinolytic activity, we downloaded all keratinase sequences from Uniprot and NCBI using keratinase as a keyword. All contigs (nucleotide) and ORFs (protein) were aligned with nucleotide and protein BLAST results (BLAST v2.6.0+) [33,34], respectively. Only hits with subject or query coverage ≥70% and identity ≥40% were retained. All reads from the poly(A)-depleted samples were also mapped to assembled bacterial genomes if a subspecies was known to possess keratinases.

### 2.6. Differential Gene Expression

Trinity’s align_and_estimate_abundance.pl script was executed to determine transcript abundance in the different samples (--est_method salmon). We then used the abundance_estimates_to_matrix.pl script (--est_method salmon, --cross_sample_norm none) to create count data for DESeq2 v1.22.2 and identify all differentially expressed contigs [41]. We compared the samples from keratin-rich and keratin-free diets, but we did not compare the poly(A)-enriched and poly(A)-depleted fractions. A contig was classified as differentially expressed if the absolute log_2_ fold-change (log_2_FC) was ≥1.5 with an adjusted *p*-value cutoff of ≤0.05.

### 2.7. GO Enrichment Analysis

All ORFs were aligned to Swiss-Prot and TrEMBL [36] using protein BLAST (BLAST v2.6.0+) [33,34] to obtain GO annotations. Hits with an e-value ≤ 0.05 were used as inputs for GO enrichment analysis. GO terms were mapped to GO slim with owltools (--map2slim) [42]. GO enrichment analysis was performed in R using the GSEABase [43] and GOstat [44] packages. The function GSEAGOHyperGParams was used with a *p*-value cutoff of ≤0.05.

### 2.8. Clustering of Contigs Containing a Trypsin Domain

To find unknown domains or sequence patterns in differentially expressed ORFs containing a trypsin domain, the ORFs were clustered using cd-hit [45,46]. The analysis was carried out three times using identity thresholds of 0.7, 0.8 and 0.9.

## 3. Results

To determine the keratin digestion mechanisms of *T. bisselliella* larvae, we compared the gut transcriptomes of larvae reared on two different diets. The keratin-rich (kr) diet was composed of goose feathers, whereas the keratin-free (kf) diet consisted of insect carcasses. We analyzed groups of larvae maintained continuously on the kr or kf diets as well as reciprocal groups that were switched between the diets (keratin-rich to keratin-free (kr2kf) and keratin-free to keratin-rich (kf2kr)) in order to detect transcriptomic changes following the dietary transition (Figure 1a). For all four diet groups, poly(A)-enriched RNA was fished from the rRNA-depleted samples, which resulted in a poly(A)-enriched and a poly(A)-depleted fraction (Figure 1b).

### 3.1. Generation of a High-Quality Larval Gut Transcriptome from T. bisselliella

We obtained 722,938,199 paired-end reads by Illumina sequencing, with an average read number of 21,262,888 ± 2,320,074 per replicate after quality trimming. Sequencing data from all rearing conditions were pooled using Trinity to construct one de novo assembly comprising 428,221 contigs on the isoform level (Supplementary Table S2) that were clustered into 285,666 pseudogenes. All further analyses were applied to the assembled isoforms, hence the term contig exclusively refers to isoform-level contigs hereinafter. The fragmentation status of the assembly was checked by mapping the sequencing reads to the assembled contigs. The overall mapping rate was 98.67%, leaving only 1.33% unmapped reads. From the mapped reads, 90.24% properly mapped as pairs.

We used TransDecoder to predict ORFs for 94,304 contigs (22.02%). Another 31.19% of the contigs were too short for analysis (<300 bp), leaving 46.79% without ORF predictions. Among the contigs with ORFs, 52.83% were classified as complete (start and stop codons present) and 47.17% as incomplete (missing start and/or stop codons) as detailed in Supplementary Table S3. To assign the assembled contigs to either the poly(A)-enriched or poly(A)-depleted fractions, reads from the two fractions were mapped separately to the assembly for exclusive assignment to one of four groups:

A. poly(A)-enriched contigs with no coverage from poly(A)-depleted samples
B. poly(A)-depleted contigs with no coverage from poly(A)-enriched samples
C. contigs covered by reads from poly(A)-enriched and poly(A)-depleted samples
D. no-coverage contigs with no coverage from either of the samples

**Figure 2:**
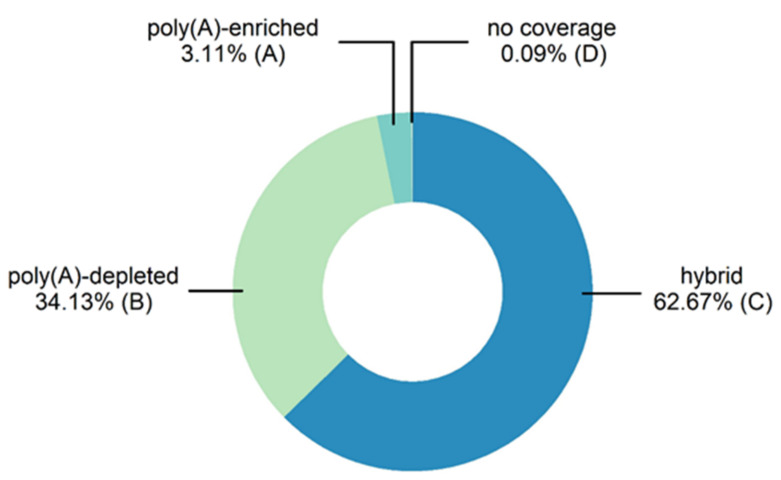
Contig classification based on read coverage. Contigs assigned to group A (poly(A)-enriched) had no coverage in the poly(A)-depleted fraction. Those assigned to group B (poly(A)-depleted) had no coverage in the poly(A)-enriched fraction. Those assigned to group C (hybrid) were covered by reads originating from both fractions, whereas those in group D (no coverage) had no coverage in either fraction.

Most of the contigs (62.67%) were covered by a combination of reads from the poly(A)-enriched and poly(A)-depleted samples and were classified into group C (Figure 2), meaning that the corresponding contigs were associated with sequence reads of both fractions. A further 34.13% of the contigs were covered exclusively by reads from the poly(A)-depleted samples (group B) and 3.11% were covered exclusively by reads from the poly(A)-enriched fraction (group A). Given the poly(A) isolation step prior to sequencing and library preparation (Figure 1b), all reads in the poly(A)-enriched fraction were considered likely to originate from eukaryotic mRNAs. Accordingly, all contigs covered by these reads, either exclusively or in combination with reads from the poly(A)-depleted fraction, were considered as eukaryotic contigs (groups A and C).

### 3.2. Taxonomic Analysis of the Larval Gut Transcriptome

Although data on the gut microbiome of the clothes moth are inconsistent, most reports could not identify abundant gut bacteria in *T. bisselliella* larvae [2,20,21,22]. We investigated the taxonomic profile of the assembly by comparing all predicted ORFs (94,304) to the UniProt database. We found that 65.59% of the ORFs had at least one hit to the superkingdom Eukaryota, 10.26% to Bacteria and 1.64% to Archaea. Furthermore, 0.12% of the ORFs had no hit on the domain level but matched other taxonomic ranks, and 0.13% were classified as viral. The remaining ORFs (22.26%) had no significant hits. Among the Eukaryota hits, the most abundant classes were Insecta (44,014 ORFs), Arachnida (4570 ORFs) and Saccharomycetes (4153 ORFs). ORFs with the best BLAST hits to Saccharomycetes may result from the preparation of the feather diet. For Bacteria and Archaea, the most abundant classes were Alphaproteobacteria (6881 ORFs) and Halobacteria (1490 ORFs), respectively. However, this analysis was only feasible for contigs with ORF predictions (only ~22% of the assembly).

We therefore analyzed the completeness of the host transcriptome and potential symbiont transcriptomes with BUSCO using all 428,221 contigs, subdivided into eukaryotic contigs (groups A and C) and poly(A)-depleted contigs (group B). Databases were selected to closely match the taxa identified during ORF classification. Optimal results were achieved when we ran BUSCO against the databases insecta_odb9 and endopterygota_odb9 for eukaryotic contigs, with 98.73% and 97.42% of the BUSCO genes found to be complete (Supplementary Table S4). For gut microbes, which could be prokaryotic or eukaryotic, optimal results were achieved when poly(A)-depleted contigs were used to screen the database clostridia_odb9 and eukaryotic contigs were used to screen the database saccharomyceta_odb9, with 23.23% and 56.51% of the BUSCO genes found to be complete, respectively. These results are described in more detail in Supplementary Table S4.

### 3.3. General Functional Annotation of the Larval Gut Transcriptome

Functional annotation of the larval gut transcriptome was achieved by screening the NCBI nr and nt databases as well as UniProt and Pfam. We assigned functions to 117,579 (27.45%) of the 428,221 contigs. From this annotated set, 81,035 contigs were previously also predicted to have ORFs (85.92% of the predicted ORFs). The remaining 36,544 contigs (no ORFs) were annotated solely using the NCBI nt database (Supplementary Table S5). For 60,341 contigs with annotations and ORF predictions, we found significant homologies to conserved Pfam domains (an average of 2.27 conserved Pfam domains per ORF) and more than half of all domains were classified as complete (Table 1).

### 3.4. Diet-Dependent Differentially Expressed Contigs

To identify diet-dependent differences in gene expression in *T. bisselliella* larvae, we compared gene expression levels for larvae grown on or transferred to the keratin-rich diet (kr and kf2kr) with larvae grown on or transferred to the keratin-free diet (kf and kr2kf), using DESeq2. The kf↔kr (↔ = comparison of the two conditions) comparison revealed 625 contigs with significant differential expression in the poly(A)-enriched samples, 360 upregulated and 265 downregulated on the keratin-rich diet (Table 2). Thereby, the 360 upregulated contigs potentially also induced those associated with keratin feeding. Accordingly, we compared poly(A)-enriched samples from the continuous keratin-free diet to those switched from the keratin-free to the keratin-rich diet (kf↔kf2kr), revealing 1622 differentially expressed contigs. The reciprocal switch was also compared to the continuous keratin-rich diet (kr↔kr2kf), revealing 808 differentially expressed contigs.

**Figure 3:**
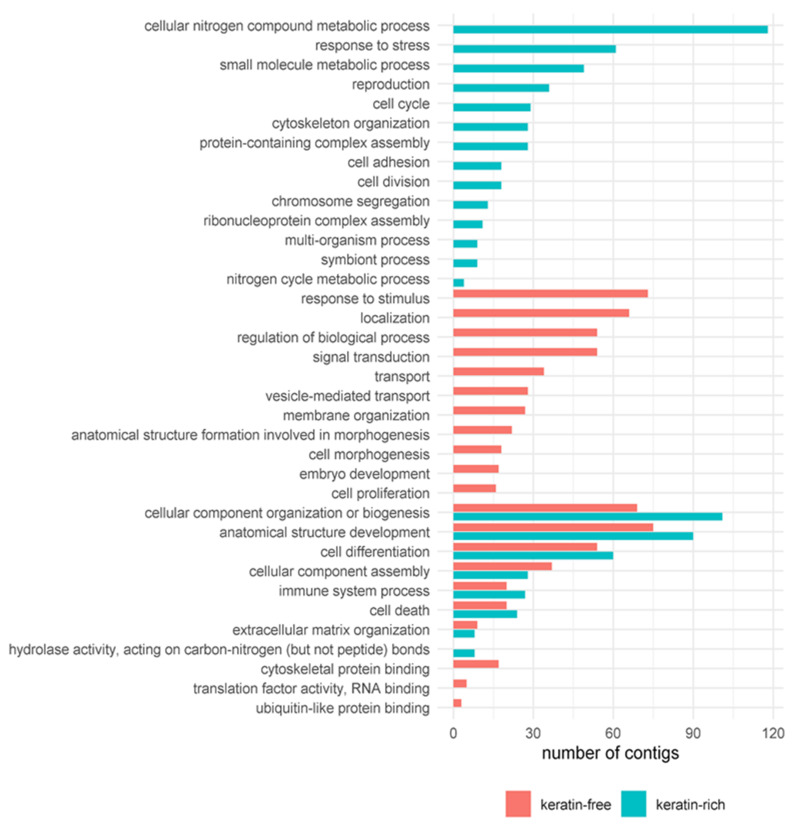
GO-Slim annotation of differentially expressed contigs from the kf↔kr comparison. Only results for the main GO domains of molecular function and biological process are shown. Red and blue bars represent contigs upregulated in the keratin-free and keratin-rich diets, respectively. The x-axis represents the number of contigs with the corresponding GO term. Bidirectional arrows (↔) show comparisons in each analysis.

We assigned GO terms to contigs with predicted ORFs, and GO enrichment analysis was applied to the differentially expressed contigs in each comparison (Figure 3). The enriched GO terms for differentially expressed contigs in the continuous keratin-rich diet (kf↔kr) included cellular nitrogen compound metabolic process (GO:0034641), response to stress (GO:0006950) and small molecule metabolic process (GO:0044281). In contrast, the enriched GO terms in the keratin-free diet were associated mainly with transport processes and proliferation, including anatomical structure development (GO:0048856), localization (GO:0051179) and transport (GO:0006810). In addition to GO terms that were enriched on only one of the diets, a few GO terms were enriched on both diets. These were mainly associated with regular cellular processes, such as component organization and biogenesis, anatomical structure development, cell differentiation, and cellular component assembly. We also identified the underrepresented GO terms in both diets, which included peptidase activity (GO:0008233) and catalytic activity acting on a protein (GO:0140096).

The comparison of GO enrichment results from differentially expressed contigs representing all four diet groups (including diet shifts) showed that most of the enriched GO terms differed between the four conditions. There was no general GO profile found for the keratin-rich or keratin-free diets. Nevertheless, the GO terms, cofactor metabolic process, sulfur compound metabolic process, transmembrane transporter activity, and nitrogen cycle metabolic process, were enriched in the kf↔kf2kr comparison.

### 3.5. The Peptidase Landscape of the Larval Gut Transcriptome

**Figure 4:**
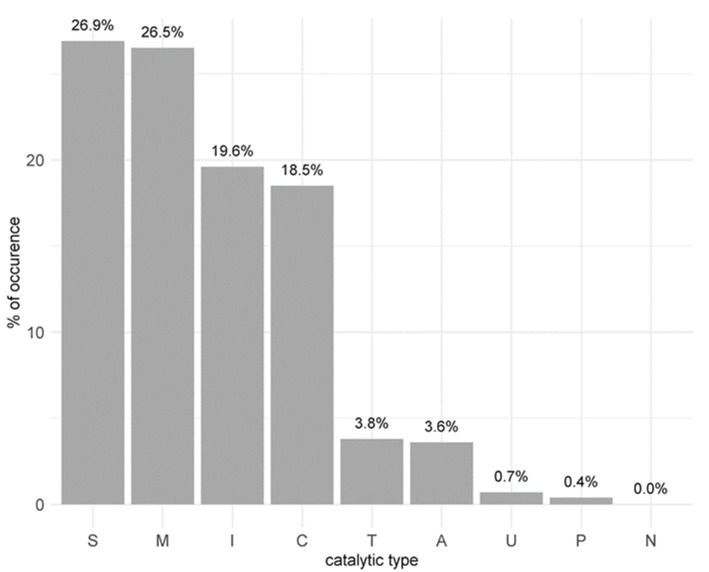
Catalytic domains of contigs with open reading frames (ORF) predictions. MEROPS annotations revealed 2613 peptidase sequences after identity filtering (query or subject coverage ≥70% and identity ≥40%). A = aspartic; C = cysteine; I = inhibitor; M = metallo; N = asparagine; P = mixed; S = serine; T = threonine; U = unknown.

Knowledge of the peptidase landscape in the gut of *T. bisselliella* larvae is limited to protein isolation studies and one analysis of ESTs [2,47,48,49]. To determine the overall peptidase composition of the transcriptome, all contigs with ORF predictions were screened against the MEROPS database. Similarities to known peptidases were found for 2613 ORFs with at least one hit against the database (e-value ≤ 1 × 10^−5^, query or subject coverage ≥70%, amino acid identity ≥40%). Among these, only 115 ORFs were expressed exclusively in the poly(A)-depleted samples, whereas the remainder were either specific for the poly(A)-enriched samples (13 ORFs) or present in both (2485 ORFs). The most common catalytic domains among these candidate peptidases were the serine-type (26.90%) and metallo-type (26.52%), whereas the least abundant were the mixed-type (0.38%) and asparagine-type (0.04%). Another 19.59% contained inhibitor-like domains (Figure 4). In contrast to previous studies, which did not predict cysteine-type peptidases [2,47,48], we identified 483 sequences (18.48%) in this group (Figure 4). Most of the ORFs were assigned MEROPS identifiers pointing to non-peptidase homologs or unassigned peptidases. The MEROPS families of these ORFs suggest associations with the ubiquitin pathway or endopeptidase activity.

GO enrichment analysis of differentially expressed contigs with ORF prediction had shown that ‘peptidase activity’ was an underrepresented category. Only 66 of the contigs annotated as peptidases with MEROPS were differentially expressed in any of the rearing conditions, but no diet-related expression of a special peptidase type was observed. Only 19 contigs were differentially expressed between the long-term diets (kf↔kr), of these, 13 were associated with the keratin-rich diet. We searched for peptidase identifiers in the other DESeq2 comparisons (kf↔kf2kr and kr↔kr2kf) and found that 10 of these 13 MEROPS identifiers were differentially expressed in the other group comparisons. They comprised three cysteine-type, two metallo-type and one serine-type peptidase and four inhibitors. None of the six peptidases is known to digest keratin, three of the inhibitors were classified as trypsin-like inhibitors, and one had no further description.

### 3.6. Detailed Screening for Potential Keratinases in the Larval Gut

Next, we screened the transcriptome data for homologies with known bacterial keratinases and identified four candidates (Table 3). Two had no ORF predictions (DN159021_c0_g1_i1, DN300764_c0_g1_i1) and were similar to the *Bacillus subtilis* strain YYW-1 KerC (kerC) gene at the nucleotide level. Although alignments for these two contigs showed a high degree of sequence identity, they only covered ~20% of their best database hit (subject coverage), which means they only represent fragments of the native full-length transcripts. The other two contigs had ORF predictions and were similar to two keratinases with amino acid identities of ~41% and ~42% identity, respectively, and query coverage ~50%. The best BLAST hit coverage of contig DN123288_c0_g1_i1 was only 36.55% and included a peptidase S8 domain fragment. The subject coverage of contig DN94215_c0_g1_i1 was 91.64%, with a best hit similar to a keratinase from *Meiothermus taiwanensis* WR-220 and regions homologous to the peptidase S8 and inhibitor I9 domains, both classified as complete. To determine whether these four contigs could influence the utilization of keratin, we also analyzed their expression levels. None of the four contigs was differentially expressed in the kf↔kr comparison for poly(A)-depleted samples.

Few bacteria possess keratinase genes and those with a sequenced genome are rare. Accordingly, a complete genome sequence was only available for *M. taiwanensis* WR-220 (A0A2H4A2Y5) and we therefore used this genome along with two others closely related to the *B. subtilis* BLAST hit and four additional genomes (three *Bacillus* and one *Clostridium*, see Supplementary Table S6), to analyze their coverage by reads from the poly(A)-depleted fraction. This remapping showed an overall low average coverage depth of 0.16-fold to 4.11-fold (only 0.16–14.48% of the genome sequences were effectively covered by reads). Results for remapping to the *M. taiwanensis* WR-220 genome, which included the most complete keratinase hit, had an average coverage depth of 0.53 and only 0.16% of the total genome was covered by reads. For all genomes, we identified peak coverage depths of 700 or more within rRNA regions.

### 3.7. Comparison of the Transcriptome with Reported Keratinase Related Enzymes of T. bisselliella

Several enzymes potentially involved in the degradation of keratin have been identified in the *T. bisselliella* larval gut. We downloaded 183 non-redundant entries from UniProt for cysteine desulfhydrase, cysteine lyase and cystine reductase [13,14,16,17] and found 39 homologous contigs in our transcriptome assembly using BLAST. Three of these belonged to the poly(A)-depleted fraction and the remaining 36 belonged to the poly(A)-enriched fraction. None of the contigs was differentially expressed in the kf↔kr comparison. Mean expression values varied from 0 to 6000. All except two contigs contained a single additional domain, the most common of which was the PALP domain.

One contig among the 36 homologous contigs from the poly(A)-enriched fraction was similar to cystathionine γ-lyase, eight resembled a bifunctional cystathionine γ-lyase/cysteine synthase and two resembled cystathionine β-synthase. Another 16 contigs were similar to cysteine synthases. All of these sequences possess desulfhydrase activity, releasing H_2_S from l-cysteine. Among the other contigs, one was homologous to a cystine reductase-like protein but the mean expression level was <1, five were homologous to (putative) cystathionine β-lyases, one was homologous to tryptophanase and two were homologous to 1-aminocyclopropane-1-carboxylate deaminase.

### 3.8. Identification of Additional Differentially Expressed Genes Potentially Involved in Keratin Degradation

We analyzed the differential expression of genes represented by poly(A)-enriched contigs, focusing on the continuous diet comparison (kf↔kr) to find novel candidate keratinolytic proteins. We identified 625 contigs with significant differential expression, 360 of which were upregulated in samples from the keratin-rich diet (Table 2). Within this group, we searched for contigs potentially involved in keratin digestion, such as peptidases, trypsin and enzymes with disulfide-reducing activity [2,50,51]. Three contigs with similarities to collagenase (UniProt) from *Papilio machaon* and *Papilio xuthus* (Lepidoptera) were differentially expressed (Table 4), two of which possessed complete trypsin domains. All three contigs had absolute log_2_FC values of 4–6, and two of them ranked among the contigs with the highest sequencing coverage (DESeq2 base mean). One of the collagenases was differentially expressed in all three DESeq2 comparisons (kf↔kr, kf↔kf2kr and kr↔kr2kf). In the kf↔kf2kr and kr↔kr2kf comparisons, four further contigs annotated as collagenases were differentially expressed and associated with keratin feeding (Table 4).

We also sought contigs with an annotated trypsin domain [2,50,51]. We found 27 such contigs differentially expressed in all three DESeq2 comparisons. Twenty of these were upregulated in at least one keratin feeding condition (continuous keratin-rich diet or switch to keratin-rich diet) but not on the keratin-free diets (Supplementary Table S7). Six contigs were upregulated in at least one keratin-free condition (continuous keratin-free diet or switch to keratin-free diet) but not on the keratin-rich diets. Finally, one contig was downregulated in the kf↔kf2kr (keratin-rich associated) and also downregulated in the kr↔kr2kf (keratin-free associated) comparisons, indicating feeding-independent differential expression. In general, differentially expressed contigs with a trypsin domain in keratin-feeding larval samples showed significantly higher mean base counts (normalized sequencing coverage) than those upregulated on keratin-free diets (Supplementary Table S7). All 27 contigs were compared to the NCBI nr database to identify the most similar BLAST hits. The twenty contigs upregulated on the keratin-rich diets were similar to collagenase and collagenase-like proteins, a serine protease, a brachyurin-like protein, mucin, chymotrypsin and hypothetical proteins, whereas the six upregulated on the keratin-free diets showed similarities to a chymotrypsin-like serine protease, a serine protease and a collagenase-like protein. The contig with diet-independent differential expression matched to an uncharacterized protein (XP_011549764.1). Sequence clustering of the 27 contigs and the analysis of their additional domains did not indicate any further diet-related classifications.

The 360 differentially expressed contigs related to the keratin-rich continuous diet included none with disulfide-reducing activities, except for two contigs similar to a γ-interferon-inducible lysosomal thiol reductase. The corresponding homologous contigs were differentially expressed in all three transcriptome comparisons and were upregulated in the keratin-rich feeding regimes.

## 4. Discussion

The clothes moth *T. bisselliella* is one of a few eukaryotic organisms known to digest keratinaceous materials. Keratin is one of the most abundant structural proteins in nature and is also a waste product from poultry farms, slaughterhouses and the leather industry [4,5]. However, the genetic basis for keratin digestion in this species is not fully understood. Thus far, only partial sequence data, such as the mitochondrial genome and a low-coverage EST dataset, are publicly available [2,52]. We therefore prepared the first de novo assembly of the *T. bisselliella* larval gut transcriptome. We provide taxonomic and functional classifications of the assembled contigs, including the gut peptidase landscape, the identity of novel keratinolytic genes, and diet-specific contig expression. Our findings will facilitate the further analysis of the *T. bisselliella* transcriptome and future molecular studies of other keratin-feeding insects. Such findings will enable a better understanding of the degradation of keratin and help to identify suitable enzymes, e.g., for the recycling of keratinous waste generated from meat and leather processing, to replace chemical treatments. In addition, keratin hydrolysate has a high nitrogen content and is rich in hydrophobic amino acids, which can be used for the production of organic fertilizers, animal feed and cosmetics [7]. The *T. bisselliella* larval gut transcriptome comprised 428,221 contigs, including sequences from *Tineola* itself and potentially others from gut microbes. The overall contig number was similar to values reported in other lepidopteran transcriptome studies, such as *Pieris rapae* [53] and *Plutella xylostella* [54]. We identified ORFs for ~22% of the contigs, which is comparable to the proportion assigned in other studies [55,56,57], although some authors have reported higher annotation rates [58]. In this context, we note that ~25% of the assembled contigs were too short for ORF prediction (≤300 bp), probably including small and non-coding RNAs and rRNA fragments left over from rRNA depletion.

Poly(A)-tail fishing was carried out before sequencing library preparation, allowing us to subdivide the total set of contigs into poly(A)-enriched and poly(A)-depleted fractions as well as hybrid contigs found in both groups. The poly(A)-enriched and hybrid (eukaryotic) contigs accounted for nearly two thirds of the total, and the rest were classified as poly(A)-depleted contigs. The latter fraction includes truncated eukaryotic mRNA, non-coding eukaryotic RNA as well as microbial sequences. The near completeness of the *T. bisselliella* assembled transcriptome was predicted based on the very high proportion of assembled reads (98.67%) and BUSCO analysis using the endopterygota and insecta datasets. The website hosting the Trinity assembly tool recommends that assemblies should be represented by at least 80% of the reads [59], and our BUSCO completeness (97.4% for the endopterygota database) was comparable to [58,60] or even higher than [61] the values reported for other lepidopteran transcriptome assemblies.

In contrast, BUSCO analysis against the microbial datasets in the poly(A)-depleted samples showed very low degrees of completeness. This might indicate a limited role for symbionts in the *T. bisselliella* larval gut, or may reflect a peculiarity of the samples we analyzed in our experiments. In line with a recent study reporting the presence of a new *Bacillus* species in the *T. bisselliella* larval gut [22], we identified a few contigs with similarities to bacterial keratinases. Further analysis of the *T. bisselliella* larval microbiome should identify the species that need to be evaluated in more detail to address the role of microbial keratinases in the dietary preferences of clothes moths.

Unspecific proteolysis is important, especially for protein degradation and amino acid recovery. Among the general class of proteases, digestive proteases catalyze the release of peptides and amino acids from dietary protein. For protein digestion, lepidopteran larvae almost entirely rely on the presence of serine proteases with differing specificities and sensitivity to inhibitors. Such enzymes have been well characterized from several different species, particularly those species which constitute major pests [62,63]. The peptidase landscape in the *T. bisselliella* larval gut has not been assessed in detail because most previous studies focused on one or a small group of enzymes [13,14,17,18,51]. We therefore screened the whole transcriptome to define a complete set of peptidases, revealing that most candidates were serine-type (703 contigs, 26.9%) and metallo-type (693 contigs, 26.5%) enzymes, with only a few representing aspartic-type, threonine-type and mixed peptidases. These findings are consistent with other studies [2,47,48]. However, those studies also reported the absence of cysteine-type peptidases, whereas we identified 483 contigs matching this category. For these contigs, missing descriptions in MEROPS did not allow us to assign a general functional activity. It may therefore be necessary to investigate the specific role of these and other cysteine-type peptidases in more detail. Although the keratin diet provides a rich nitrogen source, which is a limiting factor in many Lepidoptera, this highly cross-linked protein is inaccessible to most digestive proteases. Thus, in order to overcome this limitation and to gain access to this rich source of nitrogen, *Tineola* larvae require either a high, steady-state level of expression of keratinases, or inducible keratinases, reflecting fluctuations in the amount of accessible protein in their diet. Comparing expression levels of contigs from different diet groups, we found that most peptidases were not differentially expressed. GO analysis showed that peptidase activity is an underrepresented GO term among the differentially expressed contigs, and we were unable to assign a specific peptidase composition to one of the feeding regimes. The overall peptidase composition therefore appears diet-independent. However, we found 27 contigs homologous to trypsin that were differentially expressed and specifically associated with particular diets.

Earlier studies reported the presence of a highly active trypsin-like peptidase in the gut of *Tineola* larvae [11,47,50,51,64]. Furthermore, a crude proteinase extract with keratinolytic activity was shown to degrade ~30% of a wool substrate without the addition of a reducing agent [51]. In our transcriptome we identified 395 contigs with trypsin domains. This seems high, but multiple trypsin genes are expressed in the guts of other lepidopteran larvae, with each enzyme having different substrate specificities [65] or resistance to food-derived protease inhibitors. We also aimed to assemble all the isoforms of each gene and did not combine isoforms into unigenes, which also increased the number of contigs. Among the 395 contigs we identified, 27 showed diet-dependent differential expression, with higher expression levels generally observed on the keratin-rich diets. Clustering of the 27 contigs and domain analysis did not reveal any diet-specific characteristics. In our dataset, most of the 395 contigs with trypsin domains also contained a PALP domain, suggesting dependence on pyridoxal phosphate, the active form of vitamin B6. A lack of pyridoxine (the inactive form of vitamin B6) was shown to restrict the growth of *T. bisselliella* larvae on keratin-rich diets [10] and pyridoxal phosphate was shown to be an important cofactor for the digestion of keratin [14]. In our study, vitamin B6 was added to the keratin-rich diet by supplementing it with yeast. Yeast supplements also provide other important growth components, such as pantothenic acid, nicotinic acid and riboflavin [10]. In our study, larval growth on the keratin-free diet (insect carcasses) was not supplemented with yeast, probably explaining the observed growth delay (Figure 1a).

Interestingly, seven contigs with trypsin domains that were upregulated on the keratin-rich diet showed significant homology to collagenases from other lepidopteran species. Collagenases digest collagen, which (like keratin) is also a complex, recalcitrant, proteinaceous substrate [66]. Feathers consist of ~91% keratin, 1% fat and 8% water but no collagen [67]. Little is known about eukaryotic keratinases, but we note that many bacterial keratinases also possess collagenase activity [66]. For example, a keratinolytic protease from *Thermoactinomyces* sp. was shown to be 240-fold more active against collagen than keratin [66,68]. The candidate enzymes we identified should therefore be tested for both keratinase and collagenase activity. However, it is possible that the enzymes we have identified, including those with additional collagenase activity, are responsible for the partial degradation of keratin, even without the presence of a reducing agent [51]. It is particularly noteworthy that we did not find a general upregulation of protease-related genes on the keratin-rich diet, which would be indicative of a broad and unspecific response to e.g., low accessible nitrogen levels encountered in the diet. Instead, we identified a subset of twenty trypsin-like contigs upregulated on the keratin-rich diet, suggesting that larvae feeding on keratin can fine-tune protease gene expression. These inducible trypsins are prime candidates for the future characterization of insect-derived keratinolytic proteases.

Additional enzymes in the larval gut that may facilitate keratin degradation include cystine reductases, which reduce disulfide bonds and make the keratin polypeptide more accessible [17]. We found no contigs with similarities to a cysteine reductase, in agreement with previous experiments [14]. Cysteine lyases and cysteine desulfhydrases also possess desulfhydrase activity. However, most previous reports concerning the keratinolytic enzymes in the *T. bisselliella* larval gut were based on direct enzyme extraction and did not provide corresponding sequence information [13,14,16,17,51]. We therefore used the abovementioned enzymes as keywords for a UniProt search, and the corresponding sequences were downloaded and used for homology prediction within our contig set. In this manner, we identified three different enzymes encoded by 39 contigs: a cysteine synthase, a cystathionine β-synthase and a cystathionine γ-lyase, all of which release hydrogen sulfide from l-cysteine for the reduction of keratin disulfide bonds [13,14,18]. The *T. bisselliella* larval gut is anaerobic [69], preventing the oxidative formation of new disulfide bonds between two cysteine residues. Among the 39 contigs identified in our transcriptome dataset, the two functionally annotated as a bifunctional cystathionine γ-lyase/cysteine synthase and a cystathionine β-synthase, exhibited the highest relative expression levels. Nevertheless, the origin of the cysteine substrate for cysteine lyase/desulfhydrase activity to start the degradation process is unclear. Larval proteins may supply the cysteine [14], but it is also possible that the distinct keratinolytic activity of trypsin-like enzymes achieves sufficient keratin linearization to allow for cysteine desulfhydration and proteolysis. In support of this hypothesis, the abovementioned intestinal trypsin-like peptidases can tolerate free hydrogen sulfide [11].

## 5. Conclusions

We have constructed a high-quality transcriptome of the *T. bisselliella* larval gut. Analyses of differentially expressed contigs between the keratin-rich and keratin-free feeding regime showed that most peptidases were not differentially expressed, but we identified twenty trypsin-like contigs upregulated on keratin feeding, suggesting that the expression of these contigs was induced by the keratin-rich diet. We also confirmed the presence of previously reported enzymes and enlarged this set by adding further candidates potentially involved in the utilization of keratinous materials. Enzymes with desulfhydrase activity that may be involved in the reduction of keratin disulfide bonds are part of the *T. bisselliella* transcriptome but did not show diet-related differential expression. We also identified four contigs with homology to keratinases of bacterial origin, suggesting that bacteria may contribute to keratin digestion by *T. bisselliella* larvae.

## Figures and Tables

**Table 1:** Functional annotation of the *T. bisselliella* gut transcriptome using HMMER and Pfam.

Category	Number of Contigs
Complete	76,936
Partial	7999
Fragment	51,978

Domain annotations with a *p*-value > 0.0001 were discarded. The remaining contigs were classified exclusively into one of the three categories: complete (domain coverage ≥90%), partial (query coverage ≥90%) or fragment (all others).

**Table 2:** Numbers of differentially expressed contigs (determined using DESeq2) when comparing the two continuous diets or each continuous diet to the corresponding switched diet.

	Comparison	Differentially Expressed Contigs	Downregulated on kr Diet	Upregulated on kr Diet
poly(A)-enriched	kf↔kr	625	265	360
	kf↔kf2kr	1622	793	829
	kr↔kr2kf	808	366	442

A contig was considered to be differentially expressed at *p* ≤ 0.05 and |log_2_FC| ≥ 1.5. Abbreviations: kf = keratin-free; kr = keratin-rich; kf2kr = switch from kf to kr; kr2kf = switch from kr to kf. Bidirectional arrows (↔) show comparisons in each analysis.

**Table 3:** Best BLAST hits for contigs within the *T. bisselliella* transcriptome identified as homologs of known bacterial keratinases.

Contig	ORF pred.	Database Hit ID	Database Hit Species Name	Subject Coverage	Identity	Pfam Domain	DE
DN159021_c0_g1_i1	n.d.	EU362730.1	*B. subtilis*	19.46%	98.66% (nt)	-	no
DN300764_c0_g1_i1	n.d.	EU362730.1	*B. subtilis*	19.37%	99.10% (nt)	-	no
DN123288_c0_g1_i1	yes	A0A0H3YE38	*B. tequilensis*	36.55%	41.38% (aa)	peptidase S8 (f)	no
DN94215_c0_g1_i1	yes	A0A2H4A2Y5	*M. taiwanensis*	91.64%	42.29% (aa)	peptidase S8 (c) inhibitor I9 (c)	no

Abbreviations: n.d. = not detected; DE = differentially expressed; c = complete (domain coverage ≥90%); f = fragment (all others); nt = nucleotide; aa = amino acid.

**Table 4:** The diet-dependent expression of annotated collagenases.

	Comparison of Differential Gene Expression				
ORF (Contig Name)	kf↔kr	kf↔kf2kr	kr↔kr2kf	UniProt Entry	Pfam Domain
DN25_c0_g1_i12	+	+	+	collagenase	trypsin (c)
DN25_c0_g1_i18	+	+	n.d.	collagenase	trypsin (p)
DN3115_c0_g1_i8	+	+	n.d.	collagenase	trypsin (c)
DN2565_c0_g1_i9	n.d.	+	+	collagenase	trypsin (f)
DN2565_c0_g1_i10	n.d.	+	+	collagenase	trypsin (c)
DN25_c0_g1_i3	n.d.	+	n.d.	collagenase	trypsin (c)
DN1221_c0_g2_i2	n.d.	+	n.d.	collagenase	trypsin (c)

Abbreviations: c = complete (domain coverage ≥90%); p = partial (query coverage ≥90%); f = fragment (all others); kf = keratin-free diet; kr = keratin-rich diet; kf2kr: keratin-free switched to keratin-rich diet; kr2kf = keratin-rich switched to keratin-free diet; n.d. = not differentially expressed. The + symbol indicates keratin-associated expression. Bidirectional arrows (↔) show comparisons in each analysis.

## Data Availability

The raw sequencing reads are available in the NCBI BioProject repository with the accession number PRJNA699438. The Transcriptome Shotgun Assembly project has been deposited at DDBJ/EMBL/GenBank under the accession GJGE00000000. The version described in this paper is the first version, GJGE01000000.

## Supplementary Materials

The following are available online at https://www.mdpi.com/article/10.3390/genes12081113/s1, Table S1: BUSCO V3 databases used for BUSCO analysis, Table S2: Statistics of the de novo gut transcriptome assembly of *T. bisselliella* larvae, Table S3: TransDecoder results for the *T. bisselliella* de novo transcriptome assembly, Table S4: BUSCO summary for the transcriptome assembly of *T. bisselliella* larvae guts for different BUSCO databases, Table S5: Summary of the functional transcriptome annotation, Table S6: Genomes used as references to map reads from the poly(A)-depleted fraction against bacterial genomes, Table S7: Differentially expressed contigs of *T. bisselliella* larval guts with trypsin domains (adjusted *p*-value ≤ 0.05 and log_2_FC ≥ 1.5).
