# Supplementary material for: Next-Generation Sequencing Analysis of the Tineola bisselliella Larval Gut Transcriptome Reveals Candidate Enzymes for Keratin Digestion
Source: Genes (Basel). 2021 Jul 22;12(8):1113. doi: 10.3390/genes12081113 (PMC8394580; doi:10.3390/genes12081113)
Supplement: Supplementary file 1 [file genes-12-01113-s001.zip › genes-1304987-supplementary.pdf]

Supplementary

Supplementary Table S1. BUSCO V3 databases used for BUSCO analysis.

Database name	Download date
insecta_odb9	12 Apr 2019
endopterygota_odb9	19 Aug 2019
bacteria_odb9	16 Sep 2019
bacillales_odb9	23 Oct 2019
clostridia_odb9	23 Oct 2019
proteobacteria_odb9	23 Oct 2019
saccharomycetales_odb9	16 Sep 2019
saccharomyceta_odb9	23 Oct 2019

Supplementary Table S2. Statistics of the de novo gut transcriptome assembly of *Tineola bisselliella* larvae.

**Number of contig**	428,221
**Smallest contig [bp]**	177
**Largest contig [bp]**	50,898
**Assembled bases [bp]**	518,270,755
**Mean contig length [bp]**	1,210
**Number of contigs <200 bp**	60
**Number of contigs >1 kbp**	97,092
**Number of contigs >10 kbp**	7,404
**GC content [%]**	36.06
**N90 [bp]**	377
**N50 [bp]**	3,772

Supplementary Table S3. TransDecoder results for the *T. bisselliella* de novo transcriptome assembly.

Status	Absolute abundance	Relative abundance
complete	49,819	52.83%
5prime_partial	12,533	13.29%
3prime_partial	8,711	9.24%

internal	23,241	24.64%

Supplementary Table S4. BUSCO summary for the transcriptome assembly of *T. bisselliella* larvae guts for different BUSCO databases. [1]: Input: contigs with coverage exclusively in poly(A)dep. samples; [2]: Input: contigs with coverage in poly(A)-enriched and poly(A)-depleted samples.

**BUSCO odb9**	**Complete single [%]**	**Complete duplicate [%]**	**Fragmented [%]**	**Missing [%]**	**Total**
**Bacteria**[1]	30.41	0.68	34.46	34.46	148
**Proteobacteria**[1]	16.74	0.90	47.96	34.39	221
**Bacillales**[1]	13.11	0.57	26.24	60.08	526
**Clostridia**[1]	23.23	0.00	42.52	34.25	254
**Saccharomyceta**[2]	24.84	31.67	14.10	29.39	1,759
**Insecta**[2]	15.20	83.53	0.60	0.66	1,658
**Endopterygota**[2]	17.32	80.10	1.57	1.02	2,442

Please note that due to rounding, values may not add up to exactly 100%.

Supplementary Table S5. Summary of the functional transcriptome annotation. Peptide sequences form open rading frames (ORF) prediction were used as input for the analysis, except for the NT database, where the contigs were used as input. Percentage of ORFs was calculated with the 94,304 ORFs as base value, total number of 428,221 contigs was the base value for the calculation of contig percentage.

**Database**	**Contigs**	**ORF [%]**	**Contigs [%]**
nr	78,571	83.32	18.35
UniProt	78,859	83.62	18.42
nt	58,697	-	13.71
Pfam	60,341	63.99	14.09
MEROPS	2,613	2.77	0.61

Supplementary Table S6. Genomes used as references to map reads from the poly(A)-depleted fraction against bacterial genomes.

**Accession number**	**Reference name**
NC_000964.3	*Bacillus subtilis* subsp. subtilis str. 168 complete genome
NZ_CP048852.1	*Bacillus tequilensis* strain EA-CB0015 chromosome, complete genome
NZ_CP021130.1	*Meiothermus taiwanensis* WR-220

NC_004722.1	*Bacillus cereus* ATCC 14579, complete sequence
NC_005957.1	[*Bacillus thuringiensis*] serovar konkukian str. 97-27, complete sequence
NC_006270.3	*Bacillus licheniformis* DSM 13 = ATCC 14580, complete sequence
NZ_CP011007.1	*Bacillus pumilus*
NZ_CP011663.1	*Clostridium sporogenes* DSM795 chromosome, complete genome

Supplementary Table S7. Differentially expressed contigs of *T. bisselliella* larval guts with trypsin domains (adjusted p-value ≤ 0.05 and $log_2FC \geq 1.5$). The column "diet induced" shows whether the contig was found in samples of a keratin-rich or keratin-free diet. NR annotation column shows the best hit to NCBI nr database. complete (domain coverage ≥90%); p: partial (query coverage ≥90%); f: fragment (all others). The base mean count column shows the average of the normalized counts from DESeq2. In the absolute log_2 fold-change column the maximum log_2FC from the DESeq2 analysis is shown.

ORF ID	Diet induced	Domain status	NR annotation	Base mean count	Absolute log_2FC
DN1221_c0_g2_i2.p1	keratin-rich	complete	collagenase [*Bombyx mori*]	46096.50	3.12
DN1772_c1_g1_i6.p1	keratin-free	partial	chymotrypsin-like serine protease 4 [*Antheraea yamamai*]	12.49	3.52
DN18226_c0_g2_i5.p1	keratin-rich	complete	PREDICTED: serine proteinase stubble [*Plutella xylostella*]	23.51	4.97
DN1936_c1_g1_i3.p1	keratin-rich	fragment	serine protease 36 [*Mamestra configurata*]	39872.50	2.95
DN21748_c0_g1_i6.p1	keratin-rich	complete	serine proteinase stubble [*Bombyx mori*]	28.80	9.14
DN2565_c0_g1_i10.p1	keratin-rich	complete	PREDICTED: collagenase-like [*Papilio polytes*]	17066.42	6.01
DN2565_c0_g1_i11.p1	keratin-rich	complete	serine protease 13 [*Antheraea pernyi*]	4348.76	5.84
DN2565_c0_g1_i1.p1	keratin-rich	complete	serine protease 13 [*Antheraea pernyi*]	31751.34	3.94
DN2565_c0_g1_i3.p1	keratin-free	complete	collagenase-like [*Pieris rapae*]	935.19	3.37
DN2565_c0_g1_i6.p1	keratin-rich	complete	serine protease 13 [*Antheraea pernyi*]	211808.73	4.27
DN2565_c0_g1_i8.p1	keratin-rich	complete	serine protease 13 [*Antheraea pernyi*]	347.35	5.17
DN2565_c0_g1_i9.p1	keratin-rich	fragment	PREDICTED: collagenase-like [*Papilio xuthus*]	1661.09	4.31
DN25_c0_g1_i12.p1	keratin-rich	complete	PREDICTED: collagenase-like [*Papilio machaon*]	40378.89	6.81
DN25_c0_g1_i18.p1	keratin-rich	partial	PREDICTED: collagenase-like [*Plutella xylostella*]	43.47	4.09
DN25_c0_g1_i3.p1	keratin-rich	complete	PREDICTED: collagenase-like [*Papilio machaon*]	147.22	6.10
DN2656_c2_g1_i5.p1	keratin-free	complete	hypothetical protein B5V51_4161 [*Heliothis virescens*]	3375.24	4.11
DN2656_c2_g1_i6.p1	keratin-free	partial	hypothetical protein B5V51_4161 [*Heliothis virescens*]	10.21	3.65

DN3115_c0_g1_i8.p1	keratin-rich	complete	PREDICTED: collagenase-like [*Papilio machaon*]	7605.70	4.96
DN381_c0_g1_i3.p2	keratin-rich	fragment	serine protease [*Spodoptera frugiperda*]	204.14	2.56
DN381_c0_g1_i7.p1	keratin-rich	complete	PREDICTED: brachyurin-like [*Amyelois transitella*]	193.60	2.46
DN4341_c0_g1_i1.p1	keratin-rich	complete	PREDICTED: mucin-17 [*Plutella xylostella*]	204.68	11.52
DN568_c0_g1_i28.p1	keratin-free	complete	modular serine protease-like [*Spodoptera litura*]	40.68	23.72
DN568_c0_g1_i43.p1	keratin-rich	complete	modular serine protease-like [*Spodoptera litura*]	174.07	25.94
DN5822_c0_g1_i4.p1	keratin-free	complete	Chymotrypsin-2 [*Papilio xuthus*]	12.24	6.35
DN625_c1_g1_i3.p1	hybrid	complete	PREDICTED: uncharacterized protein LOC105381678 [*Plutella xylostella*]	9.90	-
DN7627_c0_g2_i4.p1	keratin-rich	complete	hypothetical protein B5V51_13681 [*Heliothis virescens*]	600.23	1.92
DN902_c0_g4_i1.p1	keratin-rich	complete	serine proteinase stubble-like isoform X1 [*Helicoverpa armigera*]	79.39	6.60
